# Design and implementation of a low-cost mobile robot prototype for trajectory tracking and robotic swarm tasks in research and educational applications

**DOI:** 10.1016/j.ohx.2026.e00746

**Published:** 2026-02-04

**Authors:** Donovan A. Porras Minaya, Alejandro J. Arocutipa Zambrano, Joel A. Chura, Jorge L. Huarca

**Affiliations:** Electronic Engineering Professional School, Production and Services Faculty, Universidad Nacional de San Agustín de Arequipa, Arequipa, Peru

**Keywords:** Low-cost mobile robot, Trajectory tracking, Robotic swarm, ESP-NOW, A* algorithm, Wireless communication

## Abstract

This work presents the design and implementation of a low-cost mobile robot prototype for trajectory tracking and robotic swarm tasks, aimed at research and educational applications. The robot is equipped with encoders for precise movement control and features wireless communication capabilities, enabling multiple robots to operate together using the ESP-NOW (Espressif’s proprietary wireless protocol) protocol. The input for calculating the trajectory is provided by a camera positioned 1.5 meters away, which analyzes the environment and supplies the necessary data for tracking. The system’s performance was validated through trajectory-following tests, where the robot navigated around several obstacles. The A* algorithm was implemented to calculate optimal paths and avoid collisions, ensuring smooth navigation. The results demonstrated the robot’s ability to effectively handle multiple obstacles while maintaining precise trajectory tracking. This prototype offers an affordable solution for educational and research purposes, particularly in multi-robot systems and the study of pathfinding algorithms. Future research can explore the integration of additional sensors and the optimization of the behavior of the swarm.

## Graphical abstract

**Figure:**
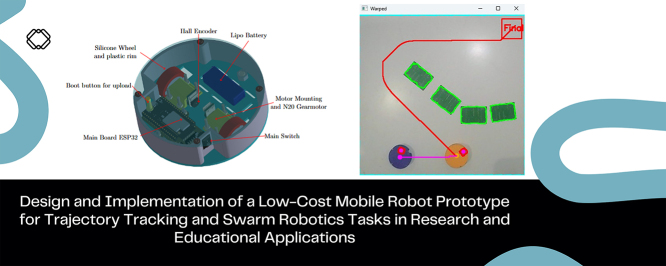

**Table:** 

**Specifications table**	

**Hardware name**	PathSwarmX

**Subject area**	•Robotics and Automation • Educational tools and open source alternatives to existing infrastructure

**Hardware type**	•Electrical Engineering and Computer Science • Mechanical Engineering and Materials Science

**Closest commercial analog**	MuSHR (Multi-agent System for non-Holonomic Racing), SMARTmBOT

**Open source license**	CC BY-SA 4.0

**Cost of hardware**	$30

**Source file repository**	DOI 10.17605/OSF.IO/S4JQF.

## 1 Hardware in context

Educational robotics has experienced significant growth in recent years, with particular emphasis on the development of accessible and modular mobile robots designed for research and teaching environments [1], [2]. These platforms provide opportunities for learning the fundamentals of robotics and exploring advanced algorithms such as trajectory planning and swarm robotics [3], [4]. Prominent examples include omnidirectional systems like *ROBOTONT*, which integrates the ROS framework [1], and modular designs like *ReFiBot*, which focus on accessibility and component reuse to foster open educational kits [2]. However, the adoption of educational robots is often limited by the high cost of commercial systems and the absence of open-source, low-cost alternatives adaptable to different academic contexts [5].

From a technical perspective, research on trajectory planning and control methods has produced a wide variety of approaches. Classical search algorithms such as A* remain widely used for efficient pathfinding and global route optimization [6], [7]. In particular, heuristic-based A* implementations offer high-quality paths in structured environments, making them a preferred choice for educational and research robots [8]. Meanwhile, adaptive and sampling-based planners enhance robustness in dynamic settings [9], [10]. Likewise, advances in robust and adaptive control have enabled cooperative formation of multi-robot systems for tasks such as exploration and collaborative automation [4], [11], [12]. In educational contexts, these methods allow students to understand trajectory optimization and coordination principles in tangible, experiment-based scenarios [13].

At the pedagogical level, the integration of educational robotics into curricula has demonstrated significant cognitive and motivational benefits. Continuous interaction with tangible robots improves visuospatial working memory, logical reasoning, and attention in primary education [14], while in higher education, robotics projects enhance engagement and active learning through experimentation [15]. Moreover, swarm robotics offers a promising framework for teaching distributed coordination and adaptive algorithms, despite the lack of standardized didactic methodologies [16]. Modular, open-source, and low-cost robots facilitate broader technological adoption by allowing customization, component reuse, and curricular adaptability [5]. These aspects contribute to the development of STEM competences and motivation across educational levels [13].

**Fig. 1:**
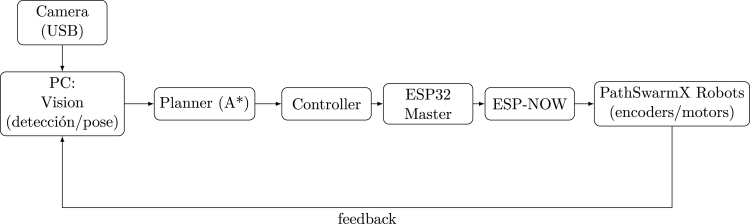
Perception–planning–control architecture with command distribution via ESP-NOW.

Finally, the integration between hardware and software has become essential for promoting computational and algorithmic thinking [17], [18]. Modern educational platforms leveraging ROS 2 incorporate state-of-the-art algorithms for navigation, planning, and obstacle avoidance [19], while visual programming environments lower technical barriers and help students focus on algorithmic logic rather than syntax [20]. The combination of sensors, actuators, and controllers fosters system-thinking skills by linking abstract algorithmic reasoning with tangible robotic behavior [21]. Furthermore, improvements in perception systems, such as fisheye camera rectification [22], and the use of algorithmic assessment metrics [23] enable objective evaluation of learning outcomes. Open-source ecosystems and rapid prototyping environments support experiential learning, encouraging students to test hypotheses, iterate algorithms, and deepen their technical reasoning [24]. The proposed architecture integrates perception, planning, and control in a distributed configuration suitable for educational swarm robotics. The overall information flow between the vision module, the planning algorithm, and the ESP-NOW network connecting multiple PathSwarmX units is depicted in Fig. 1

## 2 Hardware description

The PathSwarmX robot operates on a mobile platform equipped with motors, encoders, and an ESP32 microcontroller. A key component of the system is an external web camera, which is connected to a PC for image processing. The camera plays an essential role in enabling the robot to perform tasks such as trajectory tracking and obstacle detection.

In addition, the PathSwarmX robot is designed with simplicity and accessibility in mind, ensuring that users can easily set it up and operate it. Its architecture supports the integration of a wide range of control algorithms, including those focused on trajectory tracking, making it a versatile tool for educational and research purposes. The inclusion of the ESP32 microcontroller as the central processing unit greatly enhances its capabilities by providing Bluetooth and Wi-Fi connectivity. These features not only enable seamless communication between the robot and external devices but also facilitate the coordination and operation of multiple robots simultaneously. This makes PathSwarmX an ideal candidate for exploring swarm robotics, in which numerous units work collaboratively to perform complex tasks.

The robot’s ability to work as part of a swarm is further enhanced by the ESP32’s communication features, which allow multiple PathSwarmX robots to exchange information and synchronize their actions. This coordination enables the robots to collaborate on tasks such as search and rescue, exploration, and mapping, where collective decision-making and task division are key. The modular design of the robots also allows them to be easily adapted or expanded, creating a scalable swarm system.

### 2.1 Hardware architecture

The PathSwarmX robot is a compact and modular differential-drive mobile platform designed with simplicity, low cost, and reproducibility in mind. Its mechanical structure consists of a lightweight 3D-printed chassis that houses all electronic and electromechanical components in a layered configuration to facilitate assembly and maintenance. The primary actuation system is composed of two N20 DC gearmotors coupled with high-resolution magnetic Hall-effect encoders, which enable closed-loop control of both wheel velocity and displacement. These motors are securely mounted using custom brackets that minimize vibration and ensure precise wheel alignment.

Electrical power is supplied by a rechargeable LiPo battery, which delivers sufficient voltage and current to feed the motors, logic circuitry, and communication modules. A main power switch is installed for safe system activation, and a step-down voltage regulator ensures stable 5 V and 3.3 V outputs for the ESP32 microcontroller and peripheral devices. To facilitate firmware uploading without direct access to the ESP32 board, an external boot/reset button is routed to the chassis surface.

At the core of the system lies the ESP32 microcontroller, which integrates dual-core processing, Wi-Fi, Bluetooth, and support for ESP-NOW peer-to-peer communication. This enables real-time coordination between multiple robots without relying on external network infrastructure, which is essential for swarm robotics applications. The microcontroller also manages motor control through PWM signals, encoder feedback acquisition via interrupt-enabled GPIO pins, and communication with the host PC or other robots.

The drivetrain is completed with silicone wheels mounted on plastic rims, offering an optimal balance between traction and durability on smooth indoor surfaces. All wiring is routed through designated channels in the chassis to reduce electromagnetic interference and maintain a clean layout for educational use and replication by students or researchers.

**Fig. 2:**
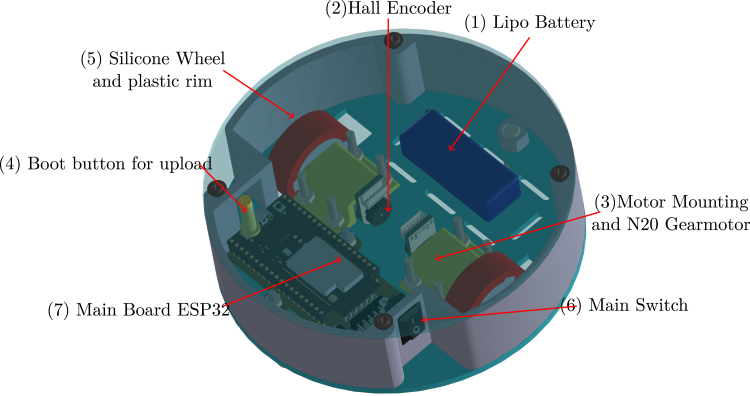
Assembled PathSwarmX robot with labeled components: (1) LiPo battery; (2) Hall encoder mounted on the N20 motor; (3) Motor mounting bracket and N20 gearmotor; (4) Boot button extension for ESP32 firmware upload; (5) Silicone wheel with plastic rim; (6) Main power switch; (7) ESP32 main board.

All these components and their physical arrangement within the chassis can be clearly observed in Fig. 2, where each element of the hardware architecture is labeled for easy identification by the reader.

### 2.2 Power supply and control electronics

**Fig. 3:**
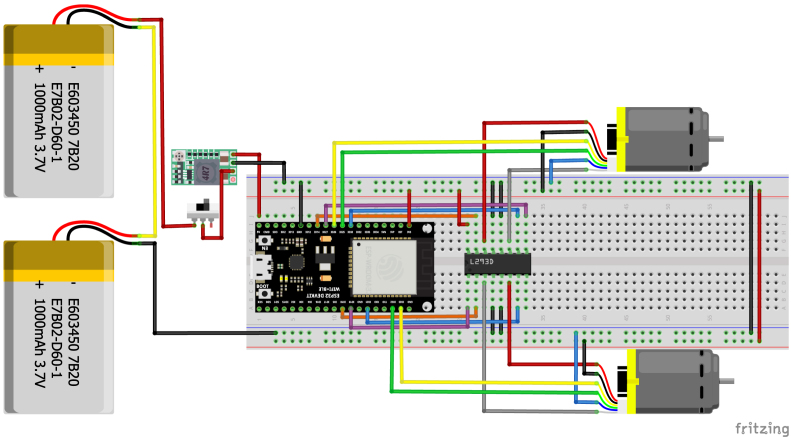
Complete circuit diagram of PathSwarmX, showing power distribution, voltage regulation, and control signal connections between ESP32, MP2314 buck converter, LM1117 regulator, L293D motor driver, and DC motors.

The complete electronic architecture of the PathSwarmX robot is illustrated in Fig. 3. The system is powered by a 2S lithium polymer (LiPo) battery rated at 7.4 V, which serves as the primary energy source for both the control electronics and the actuation units. A main power switch is placed in series with the battery to allow safe system start-up and shutdown.

To ensure safe voltage levels for all components, the battery output is fed into an MP2314 step-down (buck) converter, which regulates the voltage to a stable 5 V. This 5 V line supplies the motor driver and, in turn, is further regulated to 3.3 V by the LM1117 linear voltage regulator integrated on the ESP32 development board. This two-stage regulation guarantees a stable and noise-free power supply for sensitive control electronics such as the ESP32 microcontroller and sensor interfaces.

Motor actuation is handled by an L293D H-bridge driver. The ESP32 provides pulse-width modulation (PWM) signals to the enable pins of the L293D for speed control, while digital I/O pins define the motor direction. The H-bridge receives power directly from the 5 V line and distributes controlled voltage to the DC gearmotors, allowing bidirectional rotation.

This configuration ensures electrical isolation between logic and power domains, protects the ESP32 from voltage fluctuations caused by motor transients, and allows precise control of motor speed and direction. The resulting architecture is compact, low-cost, and robust for educational and research applications in mobile robotics.

### 2.3 Software system overview

The software architecture of PathSwarmX follows a distributed processing approach, where high-level computation is executed on an external PC, while low-level actuation and feedback are handled by embedded firmware on each robot. This separation allows the system to perform computationally demanding tasks—such as image processing, perspective correction, obstacle detection, and path planning—without overloading the microcontroller.

The PC-side software is implemented in Python and is responsible for: (i) acquiring real-time video from an overhead USB camera, (ii) applying perspective transformation to obtain a top-down orthographic view of the workspace, (iii) segmenting obstacles and detecting the pose of each robot using color-based masks and marker orientation, and (iv) generating collision-free paths using a grid-based A* algorithm. From these paths, the next motion instruction is derived for every robot in the swarm.

Instead of transmitting continuous linear or angular velocities, the master computer converts waypoint information into discrete motion commands such as “rotate left”, “rotate right”, or “advance”. These are encapsulated in lightweight packets and transmitted wirelessly via ESP–NOW to the corresponding robot. This reduces communication overhead and makes the system more robust to packet loss or latency variations, while still enabling coordinated motion.

Onboard each robot, an ESP32 microcontroller receives the command packets, interprets the requested action, and drives the DC motors using PWM control through the L293D driver. Wheel encoder pulses are read using interrupt routines and serve to verify motion execution or to detect deviations such as wheel slippage or stalling. Although no continuous velocity control is implemented in the current version, the availability of encoder data enables future use in educational settings for algorithms involving classical PID control, odometry, or fusion with vision-based localization.

This modular architecture ensures a clear division of responsibilities: the PC performs perception, mapping, and decision-making, whereas the embedded system focuses on deterministic and time-critical execution. As a result, the platform remains lightweight and low-cost, while still supporting advanced swarm behaviors and offering extensibility for research and academic purposes.

### 2.4 Perception, path planning and control pipeline

The autonomous operation of the PathSwarmX platform relies on an integrated perception-to-control pipeline that combines external vision-based localization, environment mapping, trajectory generation and decentralized motion control. Unlike conventional mobile robots that require calibrated intrinsic and extrinsic camera parameters, this system employs a planar homography-based approach. A black quadrilateral of known dimensions is placed on the workspace and detected in every frame, allowing the camera to compute a perspective transformation without manual calibration. This strategy significantly simplifies system deployment and improves reproducibility in educational and research environments.

**Fig. 4:**
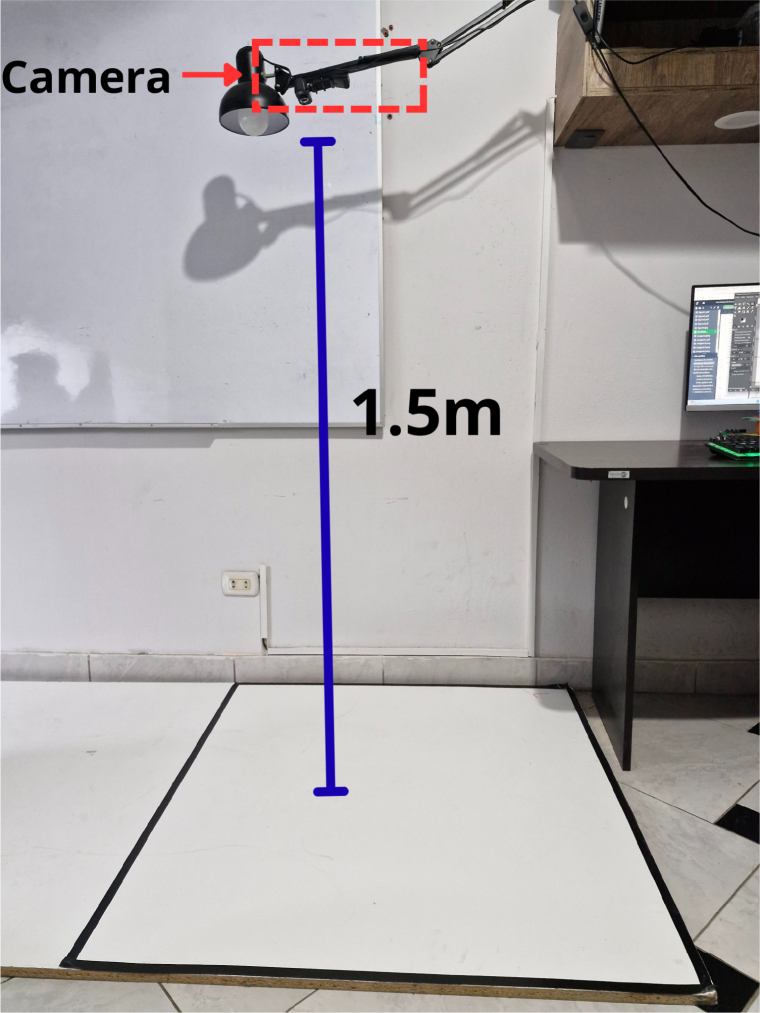
Camera positioned at 1.5 m above the workspace for global perception.

**Fig. 5:**
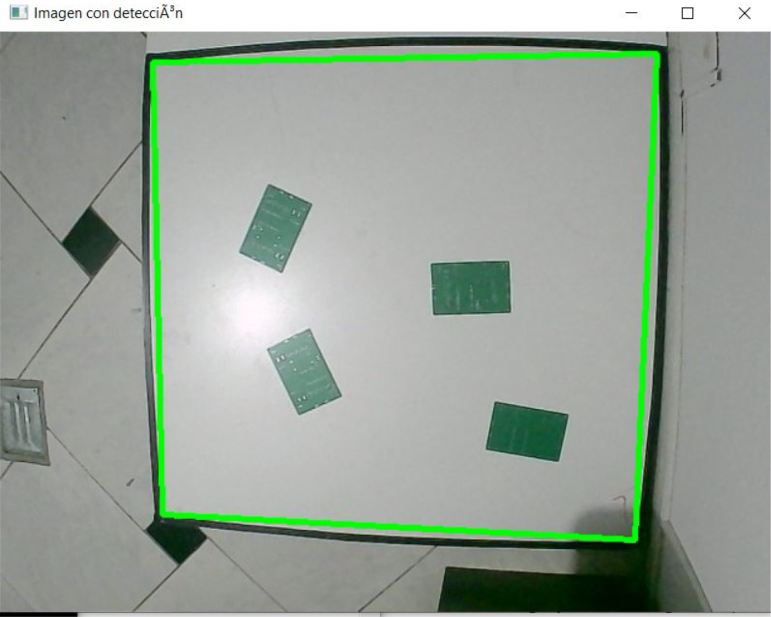
Perspective transformation result showing corrected top-down view of the workspace.

The camera is positioned at a fixed elevation of 1.5 m above the workspace to ensure full visibility of the quadrilateral reference and robot trajectories Fig. 4. The captured image is processed to detect the black reference shape, from which its four corner points are extracted. These points define a projective transformation matrix that maps pixel coordinates into real-world metric coordinates (cm), resulting in a top-down orthonormal view of the environment Fig. 5. This transformation enables accurate pose estimation of each robot and spatially consistent obstacle detection.

**Fig. 6:**
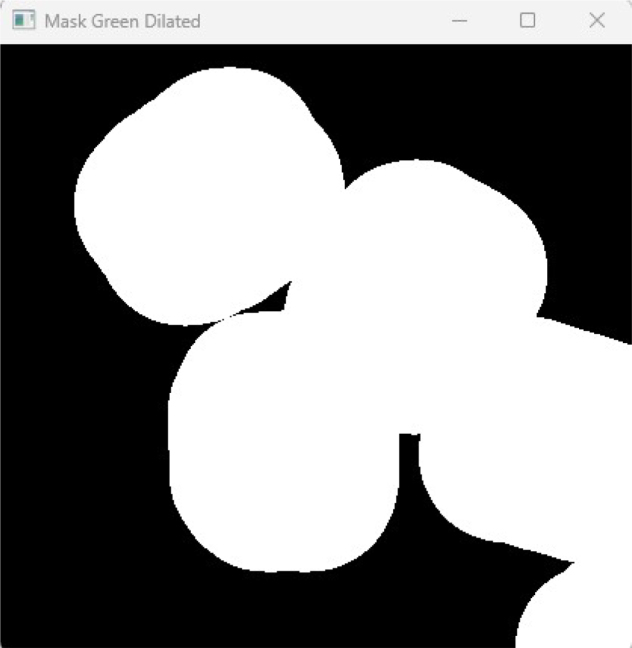
Dilated obstacle regions with 13 cm safety margin applied.

Obstacle perception is performed by applying HSV color segmentation to the orthorectified image. For the experiments presented in this work, green printed circuit boards (PCBs) were employed as obstacles; however, the system allows the use of arbitrary objects by adjusting the upper and lower HSV thresholds. A morphological dilation is subsequently applied to the binary mask using an elliptical kernel equivalent to a 13 cm safety margin Fig. 6. This ensures obstacle inflation, preventing the generation of trajectories that could lead to collisions, and guaranteeing sufficient clearance for robot passage.

**Fig. 7:**
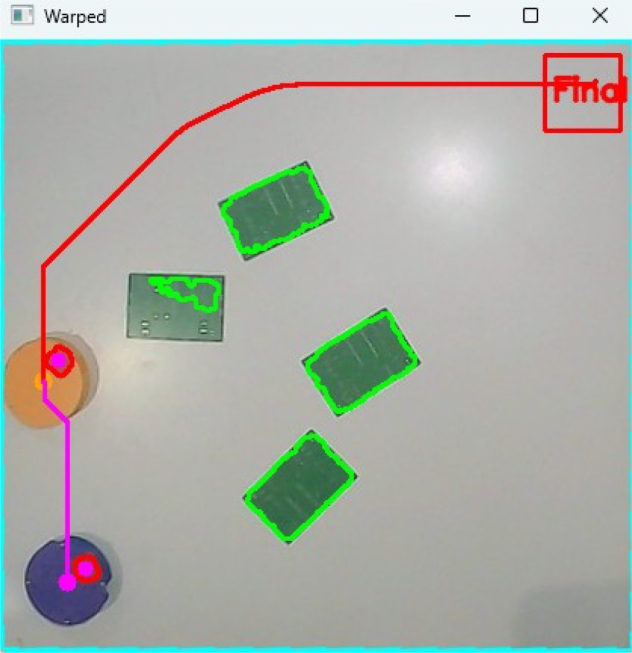
Generated paths using the A* algorithm for leader and follower robots.

Once the environment is mapped, an A* search algorithm is executed to compute optimal collision-free paths in real time. Two independent trajectories are generated: one for the leader robot, moving towards a predefined waypoint in the workspace, and a second one for the follower robot, whose objective is to reach the dynamic position of the leader Fig. 7. The paths are continuously updated as the robots move and new obstacles are detected, enabling adaptive navigation in dynamic scenarios.

**Fig. 8:**
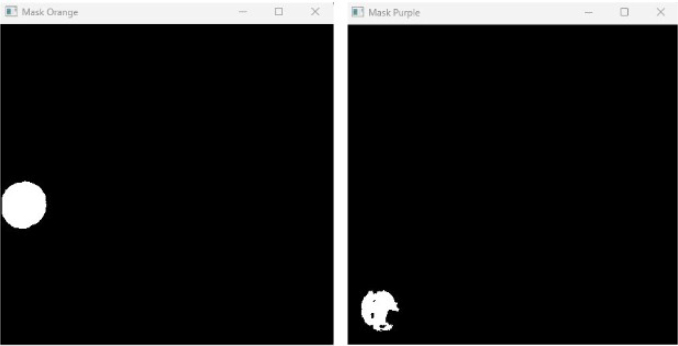
Color-based segmentation and centroid extraction for robot localization.

Robot localization is achieved by tracking the colored marker located on the top surface of each robot. The RGB image is converted to HSV, and two narrow threshold ranges are employed to isolate the leader (orange) and follower (purple). After morphological filtering, the largest connected component in each binary mask is used to compute the centroid via image moments, which yields the robot’s position in real-world coordinates Fig. 8. Orientation is obtained by detecting a square fiducial marker mounted on the robot’s frame, computing its orientation vector with respect to the centroid.

**Fig. 9:**
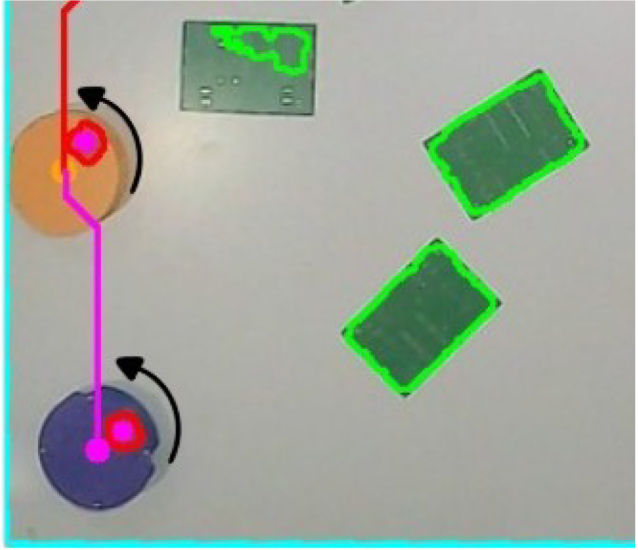
Heading alignment phase before forward motion towards the next waypoint.

The final control loop operates using a heading correction and displacement strategy. The robot is first rotated until its orientation aligns with the direction of the next waypoint. Once the heading error falls below a predefined threshold, a forward command is executed. This orient-and-move behavior is repeated for every waypoint along the A*-generated trajectory, enabling closed-loop navigation with continuous feedback from the global perception system Fig. 9.

### 2.5 Wireless communication architecture (ESP -NOW protocol)

Reliable wireless communication is essential for coordinated multi-robot operation. PathSwarmX employs the ESP -NOW protocol, a low-power 2.4 GHz communication framework developed by Espressif. Unlike conventional Wi-Fi networks—which require an access point and the overhead of TCP/IP stack management—ESP -NOW allows direct peer-to-peer data exchange between ESP32 devices without external infrastructure.

**Fig. 10:**
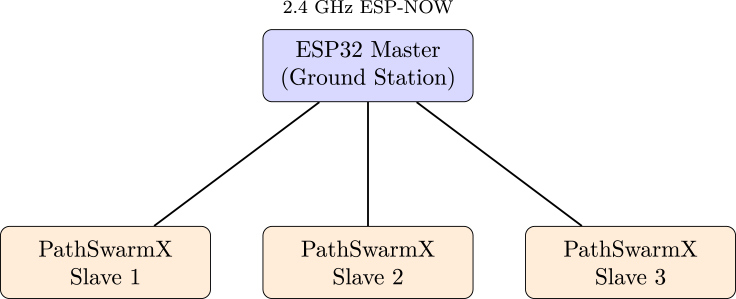
ESP-NOW peer-to-peer mesh network used by PathSwarmX. A single master node broadcasts commands and receives telemetry from multiple robots without Wi-Fi infrastructure.

In this platform, a one-to-many topology is implemented: a single ESP32, configured as the *master node* (ground station), broadcasts motion commands to all robots. Each PathSwarmX unit acts as a *slave node* and transmits only basic telemetry information (e.g., acknowledgment of received commands or encoder activity). As illustrated in Fig. 10, this results in a simple star-shaped communication architecture.

To reduce network load, each message contains only three values: {*id, action, timestamp*}. Although no formal latency measurements were conducted in this work, the system demonstrated sufficiently fast and stable communication to enable real-time control of multiple robots in practice. The use of MAC-level communication, without Wi-Fi association or routing, avoids typical delays introduced by conventional network configurations and makes ESP -NOW a practical option for small-scale swarm robotics.

## 3 Design files summary

• Base.stl: Is the main part and must be printed with more than 30 % filler because it is the support for the motors, pcb and batteries.
• Wall Robot.stl: This 3D part is used to join the part called Base.stl with the Top.stl part
• BracketN20.stl: This 3D printed part is used to attach the n20 motor to the base.
• The gerber files (.zip) are ready to be sent to be manufactured.
• The code files (.zip) it has the codes for each robot and the code for image processing and trajectory generation (see Table 2).

**Table 1:** Main hardware components and total cost of the low-cost mobile robot prototype.

Design filename	File type	Open source license	Location of the file
Base	.stl	GNU General Public License (GPL) 3.0	3D DESIGN
Top	.stl	GNU General Public License (GPL) 3.0	3D DESIGN
Rin	.stl	GNU General Public License (GPL) 3.0	3D DESIGN
Wall robot	.stl	GNU General Public License (GPL) 3.0	3D DESIGN
BracketN20	.stl	GNU General Public License (GPL) 3.0	3D DESIGN
Hold PCB	.stl	GNU General Public License (GPL) 3.0	3D DESIGN
Pulsador3D	.stl	GNU General Public License (GPL) 3.0	3D DESIGN
Circuit fritzing	.jpg	GNU General Public License (GPL) 3.0	REFERENCES
Gerber files	.zip	GNU General Public License (GPL) 3.0	PCB
Code files	.zip	GNU General Public License (GPL) 3.0	CODE

## 4 Bill of materials summary

The bill of materials provides a comprehensive summary of all components used in the construction of the robot prototype. These components were carefully selected to ensure both functionality and cost-effectiveness. The approximate price of the hardware was $30, making this solution accessible for educational and research purposes. This affordable cost is in line with the goal of promoting open-source and low-cost robotics solutions, allowing students and researchers to replicate and customize the robot for various applications. The materials used in this prototype are easily sourced, with most components available through local and online suppliers (see Table 1).

**Table 2:** Bill of materials (BOM).

Designator	Component	Number	Cost per unit - currency	Total cost - currency	Source of materials	Material type
Microcontroller	ESP32-38pin	1	$2.19	$2.19	Link	Semiconductor
H-Bridge	L293d	1	$0.099	$0.099	Link	Semiconductor
DC Motor	N20 micromotor with encoder 30:1 ratio	2	$4.39	$8.78	Link	Metal
Voltage Regulator	Step down buck type	1	$1.27	$1.27	Link	Semiconductor
Battery	Lipo battery 2S 300 mah	1	$7.65	$7.65	Link	Lithium
Wheels	Wheel N20 (optional)	2	$0.30	$0.60	Link	Plastic
Switch	Switch KBD11	1	$0.135	$0.135	Link	Plastic
Screw	Stove bolt M6X25	2	$0.35	$0.70	Link	Metal
Screw	Hex nut M6	4	$0.04	$0.16	Link	Metal
Screw	Stove bolt M3X14	12	$0.13	$1.56	Link	Metal

## 5 Build instructions

The PathSwarmX is composed of mechanical parts (body, brackets and wheels) and electronics (control board, voltage converters, encoders, motors and switch). The central unit consists of an ESP32, which runs the control software for all the modules. To manage the power supply, 5 and 3.3 V DC voltage regulators are used. In addition, the propulsion system consists of 2 motors with their respective encoders, which allow feedback for motion control and navigation. PathSwarmX is structured by the following modules:

• Control circuit and power supply in Section 5.1.
• Soldering the PCB in Section 5.2.
• Body discussed in Section 5.3.

### 5.1 Control circuit and power supply

**Fig. 11:**
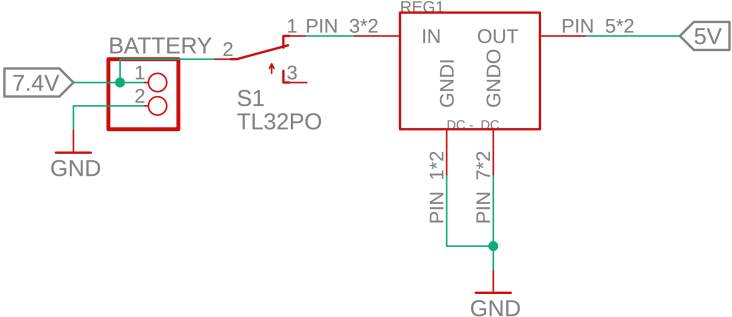
Power supply circuit of PathSwarmX.

The electronic system of PathSwarmX is composed of a power distribution stage and a control circuit. Power is supplied by a 2S LiPo battery (7.4 V), which is connected to the 2-pin male header (2.54 mm) labeled *7.4V*, as shown in Fig. 11. From there, the voltage is fed to an MP2314 step-down (buck) converter rated at 3 A, which regulates the battery voltage to a stable 5 V. This 5 V output is then delivered to the ESP32 development board, where the onboard LM1117 linear regulator generates a stable 3.3 V required for the microcontroller and logic circuitry. This dual-stage regulation ensures a reliable power supply, protecting the ESP32 from voltage drops or unexpected resets.

The PCB design files, including Gerber formats, are available in the **PCB** folder and can be sent directly to a PCB manufacturing service. This facilitates replication of the hardware and ensures consistent assembly across multiple units.

**Fig. 12:**
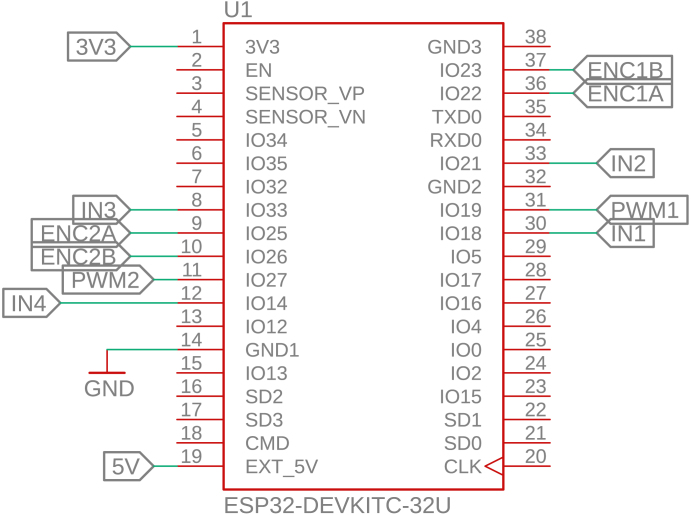
ESP32 pin configuration used in the control circuit.

The control circuit is based on the ESP32-DEVKITC-32U, which manages all software execution. Two PWM pins are used to control motor speed, while four digital outputs define the rotation direction of each motor via the L293D H-bridge driver. Additionally, four interrupt-capable GPIO pins are connected to the Hall-effect encoders, allowing real-time acquisition of motor position and speed, as shown in Fig. 12.

**Fig. 13:**
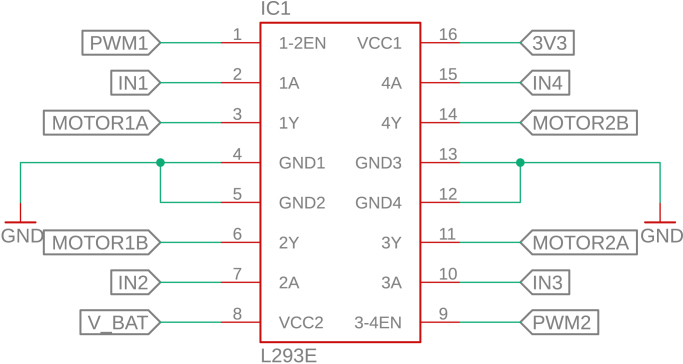
Pinout of the L293D H-bridge motor driver.

The L293D H-bridge, shown in Fig. 13, is responsible for driving the two DC motors. It supports bidirectional control and PWM-based speed modulation, supplying up to 600 mA per channel. Four logic inputs control the direction of the motors, while the enable pins receive the PWM signals from the ESP32. The driver includes thermal shutdown and current overload protection, making it suitable for low-power robotics.

**Fig. 14:**
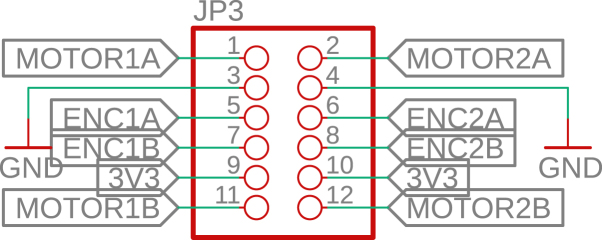
Wiring diagram of the motor encoders.

Each motor is equipped with a magnetic Hall-effect encoder. As shown in Fig. 14, each encoder contains two signal outputs (A and B) and two power supply pins. For the left motor, the pins are labeled *ENC1A* and *ENC1B*; for the right motor, *ENC2A* and *ENC2B*. These provide quadrature signals used to determine speed and direction of rotation.

### 5.2 Soldering the PCB

**Fig. 15:**
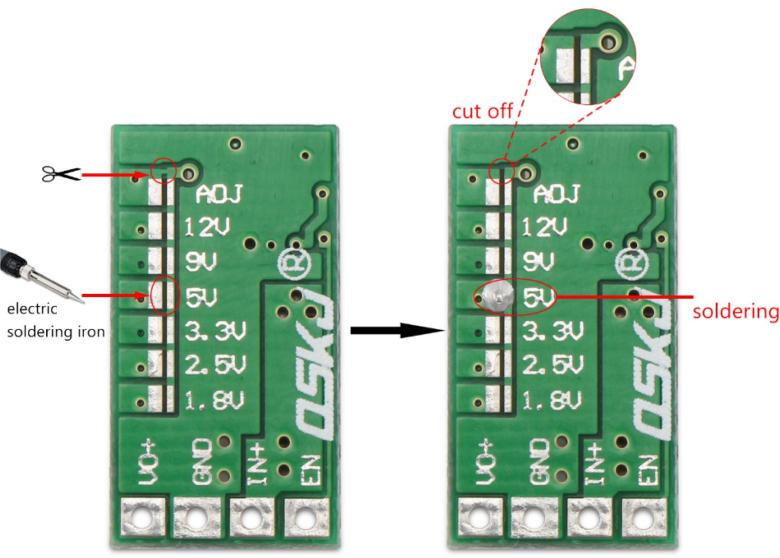
Step-down regulator with pads soldered to set the output voltage to 5 V.

To assemble the PCB, the first step is to set the buck regulator to 5 V. In Fig. 15, it is shown that the pad labeled 5 V should be soldered to establish the correct output voltage. This step is crucial to ensure that the power supplied to the system is stable and matches the required 5 V.

**Fig. 16:**
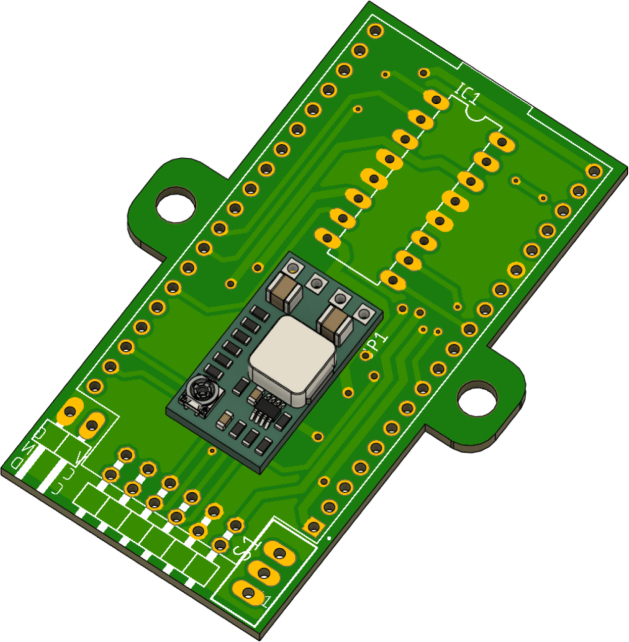
Step-down regulator MP2314 with pads soldered to set the output voltage to 5 V.

Once the 5 V regulator is set, the MP2314 module should be soldered in the area shown in Fig. 16. This module converts the battery voltage to 5 V via its built-in regulator, ensuring a stable power supply to the system. Care should be taken to properly align the input and output connections to avoid polarity reversal, which could damage the components.

**Fig. 17:**
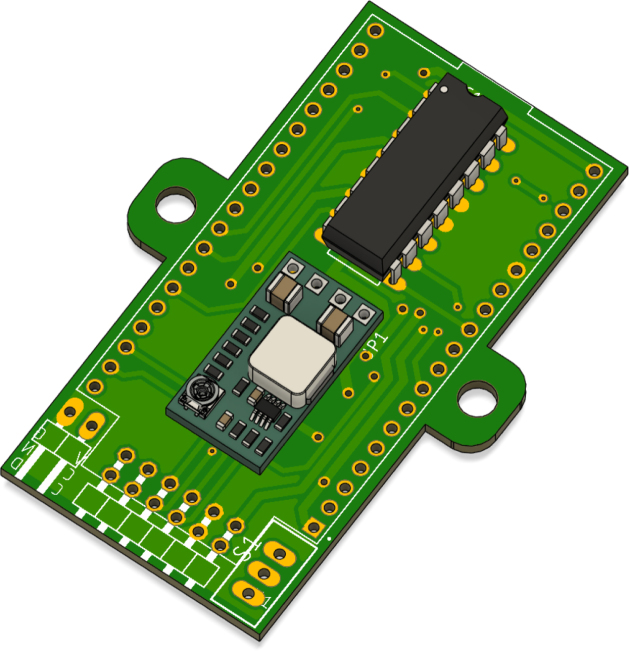
L293D H-bridge motor driver connection for bidirectional control.

Next, solder the L293D H-bridge to enable bidirectional control of the motor, as shown in Fig. 17. Correct alignment of the L293D is essential to avoid reverse operation or failure of the motors. Be sure to align the notch on the IC with the mark on the PCB to ensure proper orientation.

**Fig. 18:**
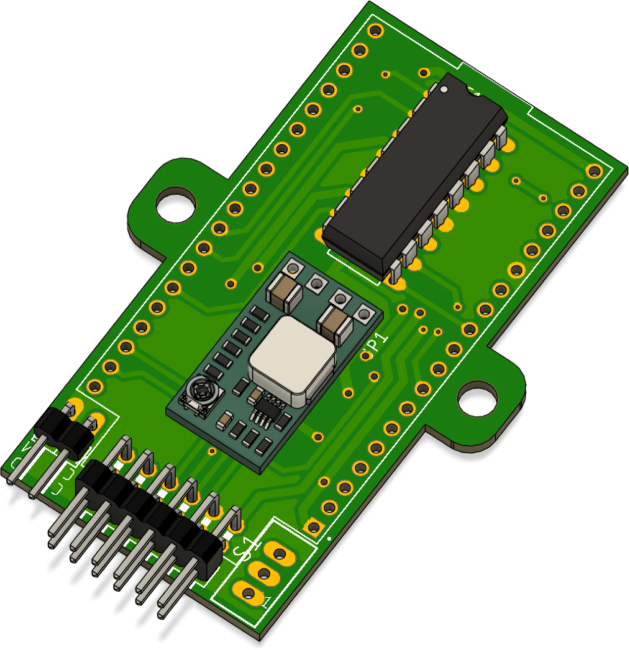
Pin headers for battery connection (2 × 1) and encoder connection (6 × 2), both 90-degree angled.

Then, solder the 2 × 1 pin header for the battery connection and the 6 × 2 pin header for the encoders, both of which are 90-degree angled headers, as shown in Fig. 18. The 2 × 1 pin header is used to connect the battery to the power supply circuit, while the 6 × 2 pin header is used for connecting the Hall-effect encoders. Proper alignment of the pin headers is crucial to ensure a solid and reliable connection. Be sure to align the pins with the corresponding pads on the PCB before soldering to avoid any misalignment.

**Fig. 19:**
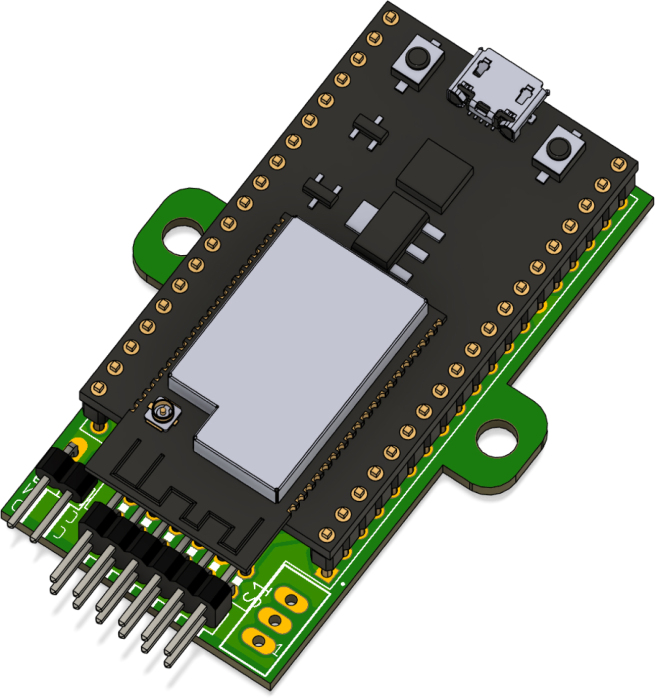
Proper orientation of the ESP32 with the USB port facing the indicated side.

To proceed, solder the ESP32, ensuring that the USB port is oriented towards the side shown in Fig. 19. Proper alignment of the ESP32 is essential to ensure that the USB port is accessible for programming and debugging. Be sure to align the ESP32 with the pads on the PCB before soldering to avoid any misalignment that could interfere with the USB connection.

**Fig. 20:**
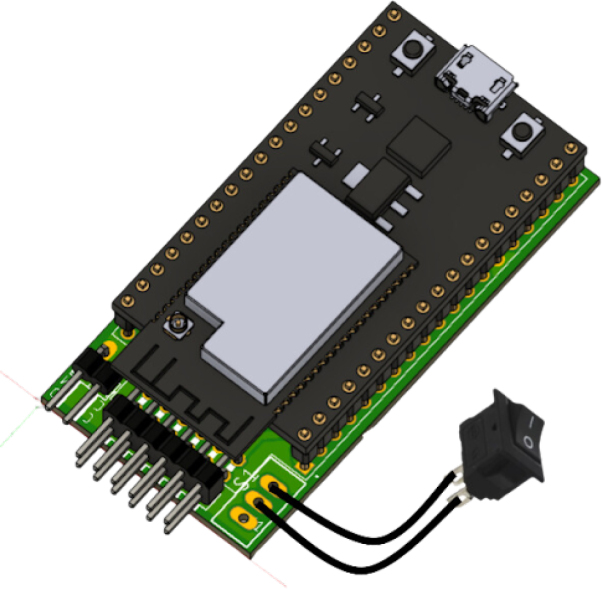
Main switch soldering location.

The main switch should be soldered as shown in Fig. 20. This component will later be embedded in the 3D-printed structure, which will be explained in further detail later on. Make sure the switch is properly aligned with the corresponding pads to ensure a reliable connection.

**Fig. 21:**
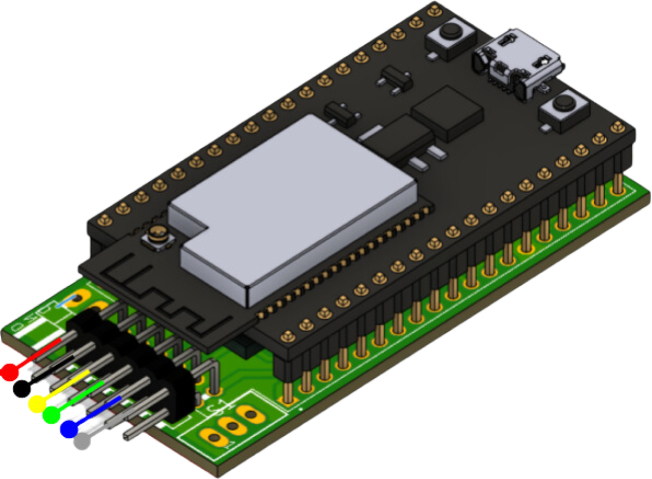
Diagram showing the wire connections for the encoder with corresponding colored lines.

It is important to clarify the order of the connections for the encoder, as it has six wires. The red wire connects to a motor pin, while the black wire is for VCC. The yellow wire is for Signal 1 of the encoder, the green wire is for Signal 2, the blue wire is for GND, and the white wire is for the motor pin. To make this clearer, I have created a diagram, shown in Fig. 21, where I have used colored lines to match each wire to the corresponding pin on the 6 × 2 male header we soldered earlier.

**Fig. 22:**
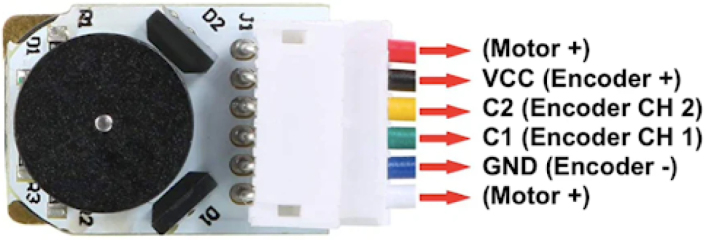
Pinout of the encoder, showing wire colors and their descriptions, as provided by the manufacturers.

Additionally, Fig. 22 shows the pinout of the encoder, with the wire colors and their corresponding descriptions, as provided by the manufacturers. This diagram will help ensure that the connections are made correctly and safely.

For the second encoder, the same operation as shown in Fig. 21 is performed, but this time on the six lower pins of the 6 × 2 male header. The wires are connected in the same order. Ensure the correct alignment and soldering of each wire to prevent any misconnection.

**Fig. 23:**
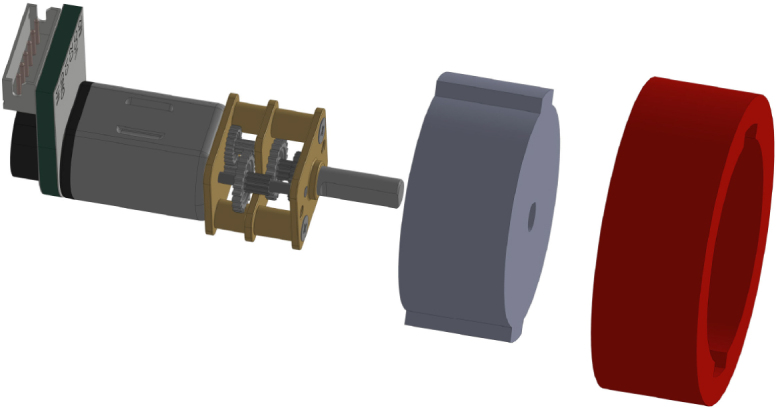
Placing the rim on the axle and mounting the rubber tire onto the rim.

After completing the encoder connections, the next step is to test the motors and verify that the encoders are working correctly. To do this, upload the encoder test code, named **encoder_test.ino**, to the ESP32. The file is located in the **Codes** folder, as indicated in the Design Files Summary table. Once uploaded, the code will print the number of steps in the serial monitor as the wheel is rotated. When rotating the wheel clockwise, the step count should increase, and when rotating counterclockwise, the step count should decrease. If the behavior is not as expected, check the wiring for any errors. Additionally, the pin assignments in the code can be modified, potentially by reversing the order of the pins, to ensure correct operation.

For proper motor operation and encoder readings, Encoder 1 is assigned to the left motor, and Encoder 2 is assigned to the right motor. When rotating Motor 1 forward (in the direction towards the battery), the step count of Encoder 1 should increment. Conversely, when Motor 1 is rotated backward, the step count of Encoder 1 should decrement. Similarly, rotating Motor 2 forward should cause the step count of Encoder 2 to increment, while rotating it backward should cause the step count to decrement.

The encoder values are printed in the serial monitor, showing the step counts for each motor. As the motors rotate forward, the corresponding encoder value should increase, and when rotating backward, the value should decrease.

This behavior ensures that the system is properly tracking the motor’s position and movement.

In the serial monitor, you will see output similar to:

**Figure:**
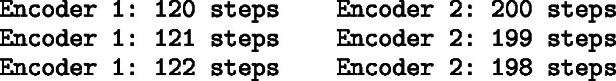

### 5.3 Body of PathSwarmX

Before starting the assembly process, all the necessary parts must be available. The PathSwarmX design consists of several elements. The main components that make up the external appearance of the robot are shown Table 3:

To begin the assembly of the wheels, the first step is to attach the rim to the motor axle, ensuring a perfect fit, as shown in Fig. 23. Once the rim is securely placed on the axle, the RTV silicone tire is carefully stretched and placed onto the rim, ensuring it fits snugly and provides even contact with the ground. Before proceeding, verify that the wheels rotate smoothly without any restrictions or excessive friction. This step is crucial to ensure proper wheel movement during operation.

**Table 3:** Mechanical components of the PathSwarmX robot body.

Components	Description
Main chassis	3D printed structural base that houses the other components
Upper deck	Top cover, protection for electronic components, also 3D printed
Side panels	Structure that reinforces the chassis and covers the connection ports
Engine mounts	Brackets that fix the position of the engines within the chassis
Motors with encoder	Elements that allow the differential traction of the robot
Rims	Parts on which the silicone tires are mounted
RTV silicone tires	Ground contact surface, providing traction
screws and nuts	M3 screws and nuts required for structural fixing
6 mm nuts	Additional support elements that stabilize the assembly

**Fig. 24:**
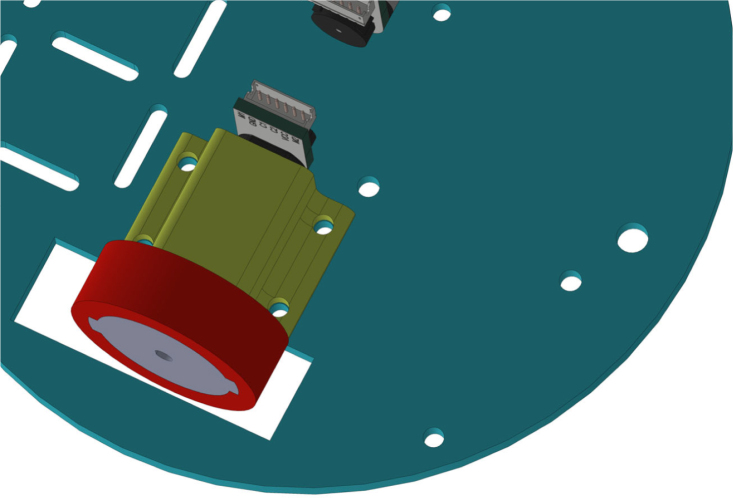
Motor mount placement on top of the motor.

The next step is to install the motor mounts. These parts are placed on top of the previously attached motors and secured with screws, as shown in Fig. 24. This step is crucial to ensure that the motor and the robot structure are firmly secured, thus avoiding unwanted movements that could affect its operation.

**Fig. 25:**
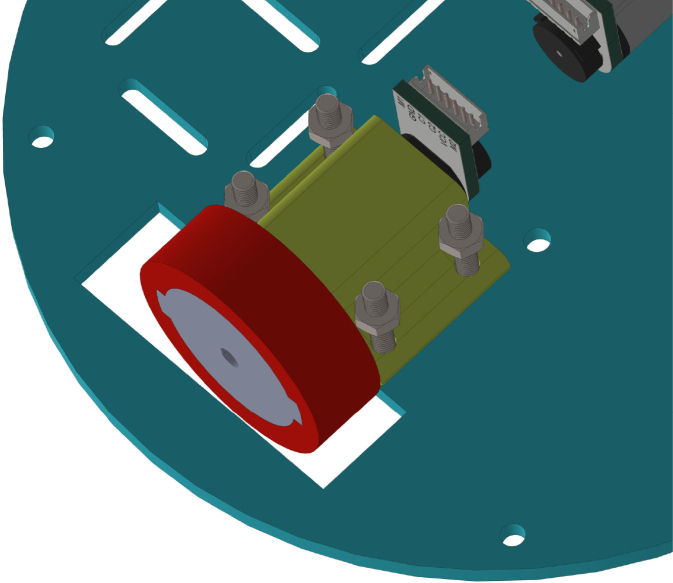
Close-up of the motor mount secured with screws.

After the motor mounts are installed, the next step is to insert the screws and nuts to secure the motor mount to the robot’s base, as shown in Fig. 25. This image highlights the process of securing the motor mount with screws and nuts, ensuring a stable installation and preventing any unwanted movement during operation.

**Fig. 26:**
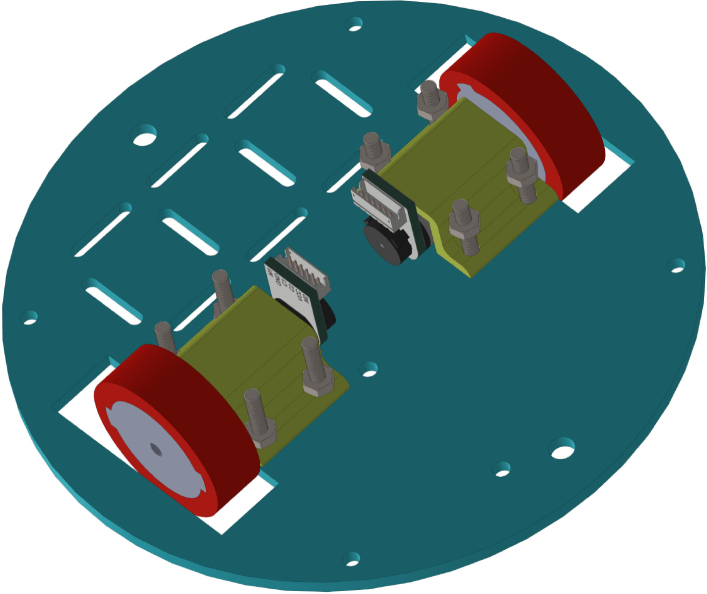
Proper placement of the two motors on the base of the robot.

In Fig. 26, the proper placement of the two motors on the base is shown, ensuring that they are correctly positioned for optimal performance and stability.

**Fig. 27:**
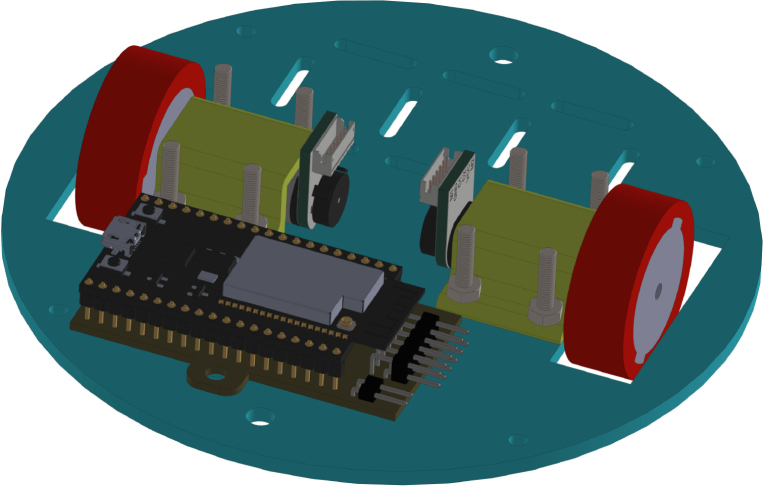
Positioning the assembled PCB on the base.

**Fig. 28:**
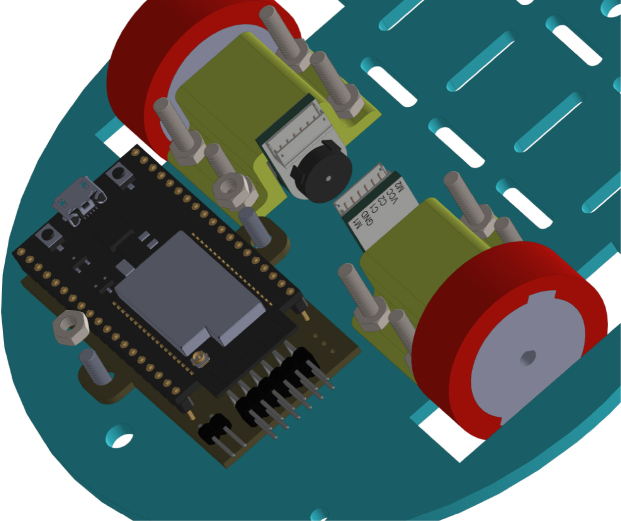
Securing the PCB to the base with 3 mm screws and nuts.

In Fig. 27, the positioning of the assembled PCB on the base is shown. In Fig. 28, it is demonstrated how the PCB should be secured to the base using 3 mm screws and nuts. The design includes two holes that align with the 3D-printed structure, ensuring a stable and secure attachment. Proper alignment and tightening of the screws are essential to ensure that the PCB remains firmly in place during operation.

**Fig. 29:**
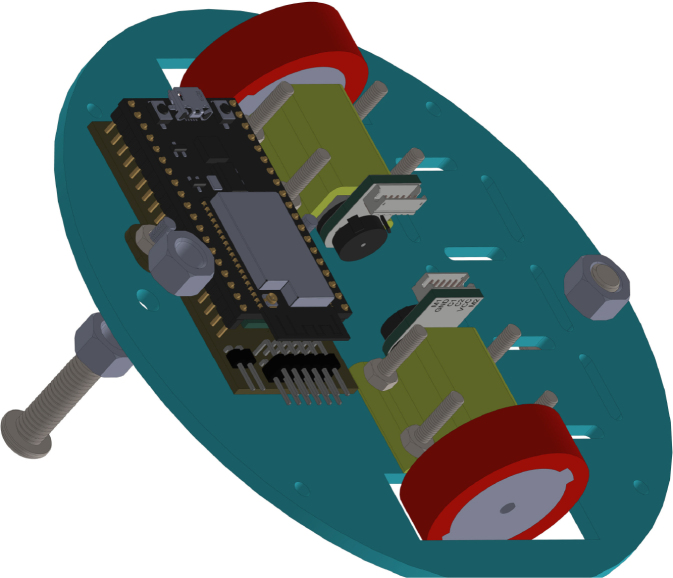
Using 6 mm screws and nuts as supports, replacing the ball caster.

As shown in Fig. 29, the next step is to use 6 mm screws with nuts to provide additional support. These screws and nuts will serve as supports for the robot, replacing the use of a ball caster. The screws should be inserted into the designated holes and tightened securely to ensure stability and proper support for the robot during operation.

**Fig. 30:**
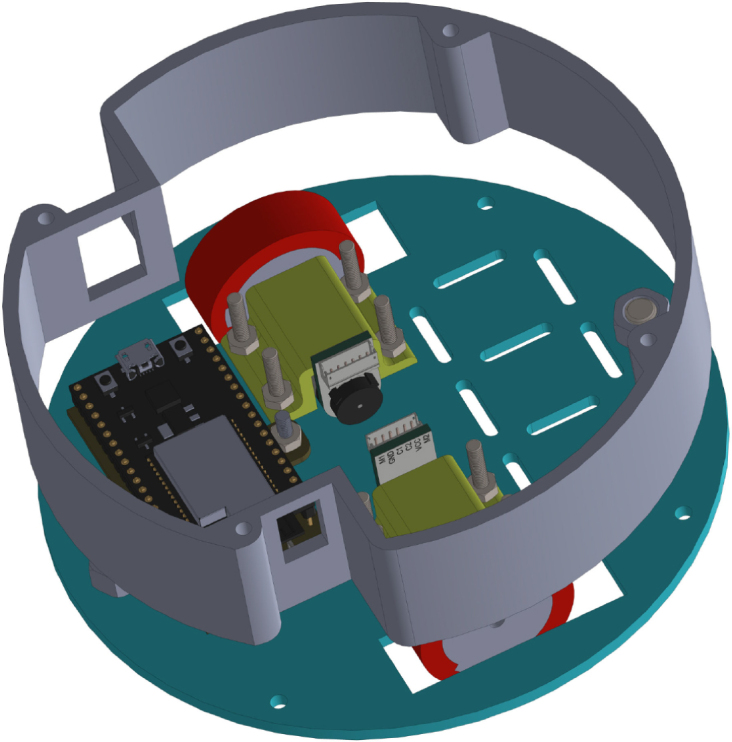
Mounting the body of the robot (wall_robot.stl).

**Fig. 31:**
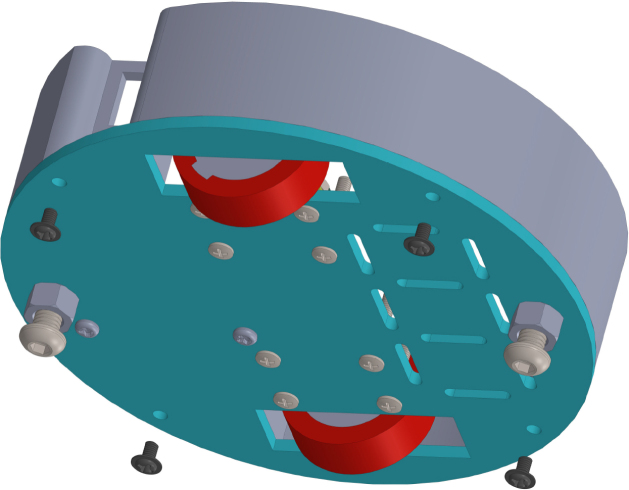
Securing the robot body with 3 mm screws.

The body of the robot should be mounted as shown in Fig. 30. This part is listed in the Design Files Summary as wall_robot.stl. After aligning the holes on the body with those on the base, secure the body by placing 3 mm screws, as shown in Fig. 31. Ensure the screws are tightened properly to provide a firm connection between the body and the base, ensuring stability for the entire structure.

**Fig. 32:**
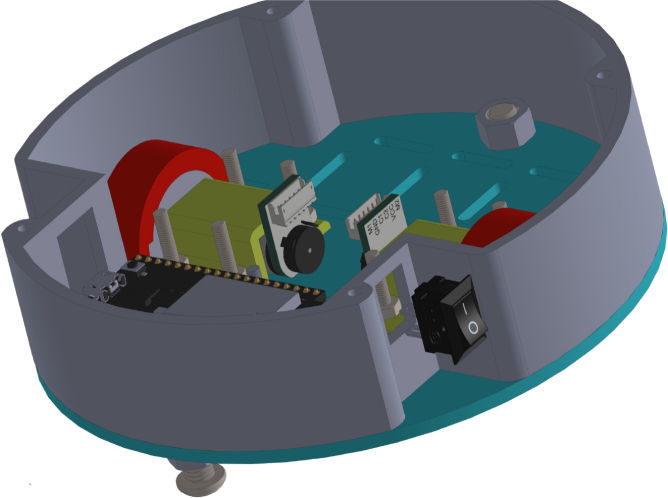
Inserting the switch into the lateral part of the robot body.

In Fig. 32, the switch is inserted into the lateral part of the robot body. Ensure that the switch is properly aligned with the designated slot on the body, as shown in the figure. The switch should be securely placed to allow easy access to turn the robot on and off. Once the switch is in place, make sure that it is firmly attached to avoid unwanted movement during operation.

**Fig. 33:**
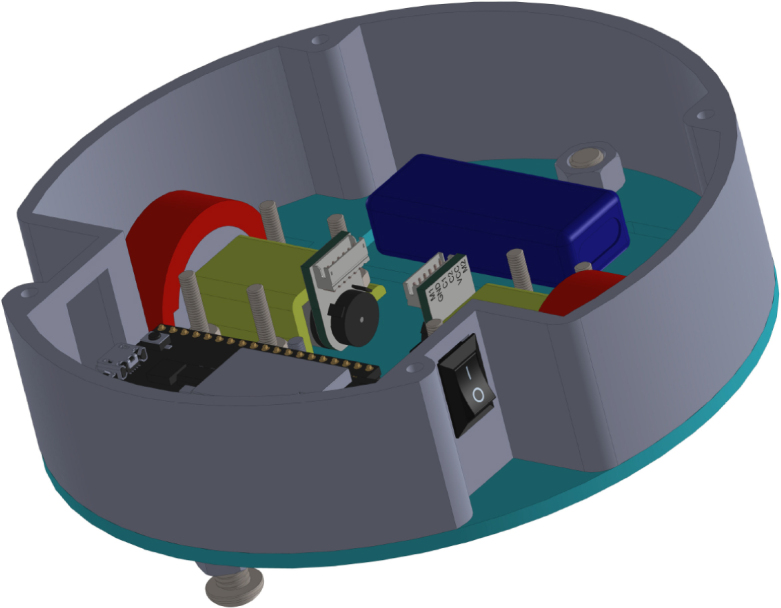
Fixing the LiPo 2S battery to the base of the robot.

In Fig. 33, the LiPo 2S battery is fixed to the base of the robot. Carefully place the battery in the designated compartment on the base, ensuring that the battery is securely positioned and properly aligned. The battery should be fastened in place to prevent any movement during operation. Proper securing of the battery is crucial for the safe operation of the robot and to avoid any disconnections or short circuits during movement.

**Fig. 34:**
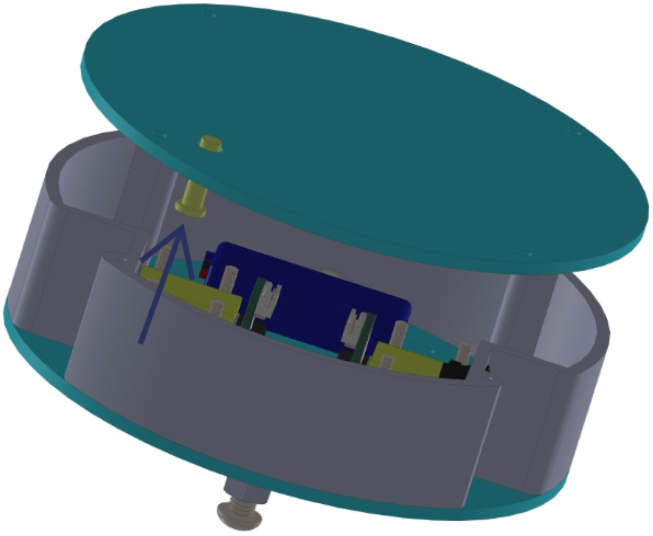
Placing the 3D-printed piece for pressing the boot button of the ESP32.

The small 3D-printed piece, found in the **3D DESIGN** folder and named **pulsador3D.stl**, is placed as shown in Fig. 34. This part is designed to allow easy access to the boot button of the ESP32 when the robot is closed. The purpose of this piece is to facilitate pressing the boot button while uploading the code to the ESP32 via Arduino, which is necessary in some situations.

**Fig. 35:**
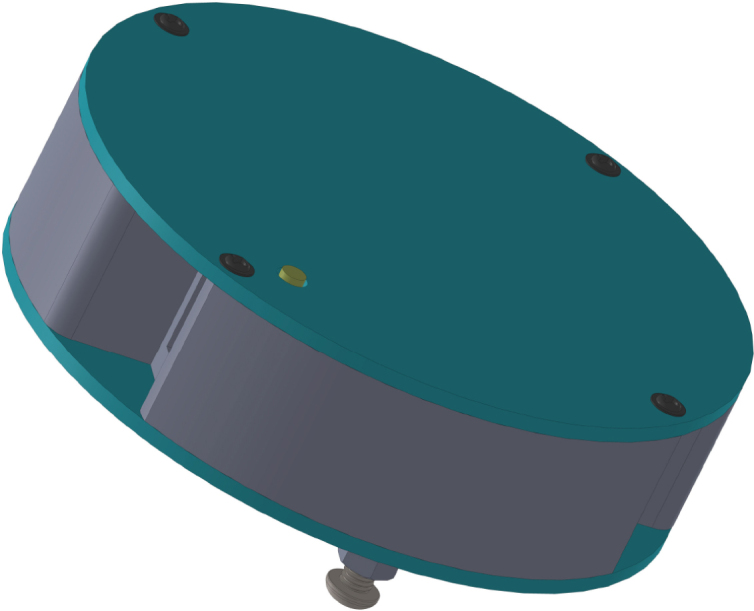
Positioning the top cover and securing it with 3 mm screws.

Finally, the top cover, labeled **Top.stl**, is positioned and secured with 3 mm screws, as shown in Fig. 35. The screws should be tightened properly to ensure the top is securely attached to the body of the robot.

## 6 Operation instructions

### 6.1 Safety concerns

Before operating the PathSwarmX system, users must ensure that both hardware and software components are correctly configured and that the test environment is prepared for safe operation. The robot operates on low-voltage electronics (7.4 V LiPo battery and 5/3.3 V regulation stages), but care should be taken to avoid short circuits or reverse polarity during wiring and battery connection. The following guidelines are recommended to prevent damage or unsafe conditions:

• **Power safety:** Always verify correct polarity before connecting the 2S LiPo battery to the power header. A reverse connection may damage the ESP32 board or the voltage regulator. Avoid operating the robot while charging the battery.
• **Electrical hazards:** Do not manipulate the PCB or wiring while the system is powered on. Disconnect the main switch before making any hardware adjustments, particularly when testing new motor drivers or encoders.
• **Thermal safety:** The MP2314 step-down converter and the L293D H-bridge may heat up during continuous operation. Avoid prolonged tasks exceeding 10 min at full load without ventilation. Allow the circuit to cool before further testing.
• **Mechanical safety:** The 3D-printed chassis and motor mounts are lightweight and not designed to withstand heavy impacts or drops. Operate the robot only on flat indoor surfaces. Avoid collisions with rigid obstacles that could misalign the wheel encoders or damage the frame.
• **Workspace preparation:** Ensure that the test area is clean, flat, and clearly delimited within the camera’s field of view. The overhead USB camera should be placed approximately 1.5 m above the workspace, ensuring stable support to prevent movement or vibration.
• **Software setup:** Always verify that the calibration of the homography transformation is correct before running trajectory or swarm experiments. Incorrect calibration may cause the robots to move beyond the intended boundaries.
• **Wireless operation:** The ESP–NOW network should be configured before powering the robots. Avoid adding new devices during execution, as it may interrupt synchronization and cause erratic motion.

These safety considerations ensure that PathSwarmX can be operated repeatedly in classroom or laboratory environments without damaging components or introducing unnecessary risks. Users are encouraged to supervise the operation during all tests and to maintain a safe distance from the robots while moving.

### 6.2 Operation instructions

The operation of PathSwarmX involves three main stages: system initialization, calibration and vision setup, and execution of motion commands. The following procedure describes the recommended steps for safe and effective use:

1. **Hardware setup.** Place each PathSwarmX robot within the workspace and verify that the wheels rotate freely. Switch on each robot using the main power switch. Confirm that the ESP32 board LED indicators are active and that the power supply delivers stable 3.3 V and 5 V outputs.
2. **Camera positioning and calibration.** Mount the USB camera approximately 1.5 m above the workspace, centered on a black 100 ×  100 cm reference square. Launch the Python vision interface and run the calibration routine to compute the homography matrix. The program will display a top-down orthographic projection used for path planning and obstacle segmentation.
3. **Network initialization.** Connect the host computer to the ESP–NOW master node (ESP32 configured as the controller). The master automatically establishes peer-to-peer links with all available slave robots. Each robot acknowledges the connection via a short LED blink sequence. If any unit does not respond, verify that its MAC address is registered in the master’s firmware.
4. **System verification.** Execute the test_encoder.ino scripts provided in the source code package to confirm correct motor polarity and encoder readings. The terminal should display incremental encoder counts during wheel rotation. Any reverse behavior can be corrected by swapping the motor polarity or encoder channels.
5. **Running the perception and planning module.** Start the main control script (main_vision_planner.py). The software will capture the camera feed, perform obstacle segmentation, and identify each robot by color. The detected workspace, obstacles, and planned paths are displayed in real time on the GUI. The user may assign target positions or activate pre-defined trajectories.
6. **Trajectory execution.** Once the path is generated by the A* planner, the master transmits discrete motion commands (*rotate left, rotate right, advance*) via ESP–NOW. Each PathSwarmX unit executes the command through PWM motor control and encoder feedback verification. During this process, the GUI updates the robots’ positions continuously to visualize progress.
7. **Swarm coordination.** For multi-robot experiments, one robot can be designated as the leader and others as followers. The follower robots automatically generate their trajectories based on the leader’s position, maintaining formation while avoiding collisions. Ensure that each robot uses a unique color marker and identifier.
8. **End of operation.** After completing the trajectory, the robots return to an idle state and await further commands. Turn off the robots using the main switch, then close the GUI application and disconnect the USB camera. Finally, disconnect the LiPo batteries for charging and storage.

Following this procedure guarantees consistent operation of the PathSwarmX platform during laboratory sessions and minimizes the likelihood of communication errors or mechanical failures. The open-source nature of both hardware and software allows students and researchers to extend the experiments by modifying the control algorithms, implementing PID or odometry modules, or integrating additional sensors for advanced research.

## 7 Validation and characterization

The experiments carried out in this section are directly aligned with the main objective of the article, which is to demonstrate that the proposed low-cost PathSwarmX platform is capable of performing autonomous navigation and coordinated multi-robot behavior with sufficient precision for educational and research applications. Therefore, rather than serving as isolated demonstrations, the tests were explicitly designed to validate the three core contributions of this work:

• **Path Planning Capability:** To verify that the perception and A* path-planning pipeline can generate feasible, collision-free trajectories using only a global overhead camera and ESP–NOW communication, without relying on external infrastructure or computationally expensive onboard processing.
• **Navigation and Control Accuracy:** To assess whether the robots are capable of physically following the planned paths and reaching target destinations with acceptable accuracy, thus validating the control strategy and the feasibility of the proposed hardware–software architecture.
• **Swarm Coordination and Timing:** To evaluate the synchronous behavior between the leader and the follower robot—critical for swarm robotics—by analyzing whether both agents can generate their respective paths, avoid obstacles, and complete their trajectories within coherent time intervals.

Accordingly, every experiment presented in this section contributes to validating one or more of these aspects. The following subsection details the evaluation of path generation and movement accuracy for both robots.

### 7.1 Validation of path generation and robot movement accuracy

The validation of the path generation system and the movement accuracy of the leader and follower robots was performed through a series of tests in which both the results of the generated path and the arrival times of each robot at its destination were analyzed.

To evaluate the path generation, a comparison was made between the paths generated by the robots. In each test, it was verified whether the leader and follower robot correctly generated the path to its destination. This was based on visual analysis of the generated paths, observing whether the robots followed the correct path and avoided obstacles correctly.

For obstacle detection, a camera was used to provide real-time information about the presence of obstacles in the environment. The camera detected the obstacles, and it was verified whether the robot navigation system allowed the robots to avoid the areas occupied by these obstacles while moving towards their destinations. Obstacle detection was considered successful if the robots could correctly navigate while avoiding the obstacle areas, which was reflected in the corresponding column of the table.

Arrival times were measured with a stopwatch, recording the time elapsed from the start of the trajectory until the robot reached the destination. The times were recorded for both robots: the leader and the follower, in each of the test cases. These times were critical to evaluate the efficiency and synchronization of the robots in following the generated trajectory.

In Table 4 and In Table 5, the results of these tests are presented as follows:

• **Image of the Generated Path**: Images of the path generated in each test are shown.
• **Path Generation**: Indicates whether the path generation was correct or incorrect for the leader and follower robots.
• **Obstacles Detected**: Reports whether the obstacles were correctly detected during the path, according to the camera.
• **Leader Time (s)**: Shows the time it took the leader robot to complete its path to the destination.
• **Follower Time (s)**: Shows the time it took for the follower robot to complete its path to the leader.

Table 6 summarizes the twelve path-generation trials and highlights three clear patterns:

**Table 4:** Path–generation results (rows 1–6).

**Table 5:** Path–generation results (rows 7–12).

**Table 6:** Overall results for the twelve path–generation tests.

Metric	Value	Comment
Total tests executed	12	Rows 1–12
Leader: path generated correctly	5/12 (41.7 %)	Rows 1, 3, 5, 7, 9
Follower: path generated correctly	12/12 (100 %)	Follower never failed
Obstacles detected by vision	8/12 (66.7 %)	Rows 4, 6, 10, 12 report “No”
Tests aborted (*time*= 0 s)	3	Rows 4, 10, 12—no obstacle mask and leader path incorrect
Mean travel time (obstacle =*Yes*)	Leader 18.8 s; Follower 22.7 s	Averaged over 8 valid runs
Mean Leader–Follower time gap	3.9 s	Follower consistently trails leader
Fastest successful run	Row 1: 15.2 s/18.5 s	Both robots correct, obstacles detected
Slowest successful run	Row 11: 24.7 s/29.4 s	Leader path incorrect on first attempt, recovered later

1. **Asymmetric performance between robots.** The follower generated its path correctly in 100 % of the cases, whereas the leader succeeded in only five out of twelve attempts (41.7 %). This indicates that the follower’s initial-orientation routine or square-marker detection is more robust than that of the leader.
2. **Critical dependence on the obstacle mask.** Every aborted run (*time* = 0 s) coincides with a failure in obstacle segmentation. When the camera cannot identify the mask, the leader loses its reference and halts the mission, suggesting the need for a fallback mechanism—e.g., a “stop-and-scan” mode—before canceling the cycle.
3. **Consistent time gap.** In the eight valid trials (obstacles = “Yes”), the leader reached the goal in an average of 18.8 s, while the follower required 22.7 s, yielding a mean delay of 3.9 s. This offset aligns with the formation strategy in which the follower avoids interfering with the leader, although it could be reduced by slightly increasing the follower’s cruise speed or shortening its waypoint look-ahead distance.

Overall, the results confirm the reliability of the vision channel for the follower and the detection of most obstacles, but also expose the leader’s susceptibility to occasional planning failures and the system’s overall sensitivity to obstacle-mask segmentation.

Beyond validating the technical feasibility of the platform, these results demonstrate that PathSwarmX is suitable as an educational tool across different academic levels. At the undergraduate level, the system enables hands-on experiments related to: (i) camera calibration and homography-based workspace transformation, (ii) HSV color segmentation and obstacle detection, (iii) A* path planning on occupancy grids, and (iv) discrete motion control using PWM and H-bridge drivers. Meanwhile, in more advanced or graduate-level courses, the same platform can be used to explore: (i) encoder-based odometry and error accumulation, (ii) cooperative leader–follower coordination and formation maintenance, (iii) perception failures and recovery strategies, (iv) communication latency and packet handling using ESP–NOW, and (v) integration of hybrid vision–odometry control loops.

In total, the architecture supports at least 6–8 structured laboratory sessions or research projects. These include calibration of the perception system, implementation of obstacle inflation via morphological operations, waypoint tracking, encoder verification, swarm coordination, and failure analysis during mask loss or planning errors. Therefore, in addition to demonstrating feasible autonomous navigation, PathSwarmX contributes as a replicable, low-cost experimental platform for teaching core concepts in robotics, perception, path planning, and multi-agent control.

## CRediT authorship contribution statement

**Donovan A. Porras Minaya:** Writing – original draft, Software, Methodology, Investigation, Conceptualization. **Alejandro J. Arocutipa Zambrano:** Writing – review & editing, Writing – original draft, Visualization, Validation. **Joel A. Chura:** Writing – review & editing, Investigation, Formal analysis, Data curation. **Jorge L. Huarca:** Writing – review & editing, Supervision, Project administration, Funding acquisition.

## Declaration of competing interest

The authors declare that they have no known competing financial interests or personal relationships that could have appeared to influence the work reported in this paper.
